# Paeonol, a Major Compound of Moutan Cortex, Attenuates Cisplatin-Induced Nephrotoxicity in Mice

**DOI:** 10.1155/2013/310989

**Published:** 2013-09-19

**Authors:** Hyojung Lee, Gihyun Lee, Hyunseong Kim, Hyunsu Bae

**Affiliations:** Department of Physiology, College of Korean Medicine, Kyung Hee University, 1 Hoeki-Dong, Dongdaemoon-gu, Seoul 130-701, Republic of Korea

## Abstract

Cisplatin is an effective chemotherapeutic agent that is used for the treatment of a variety of cancers; however, its nephrotoxicity limits the use of this drug. In the present study, we examined whether paeonol, a major compound of Moutan Cortex, has protective effects on cisplatin-induced acute renal failure in mice. To accomplish this, Balb/c mice (6 to 8 wk of age, weighing 20 to 25 g) were administered, Moutan Cortex (300 mg/kg) or paeonol (20 mg/kg) once a day. At day 4, mice received cisplatin (30, 20, or 10 mg/kg) intraperitoneally. The paeonol-treated group showed marked attenuation of serum creatine and blood urea nitrogen levels as well as reduced levels of proinflammatory cytokines and nitric oxide when compared to the control group. In addition, the paeonol-treated group showed prolonged survival and marked attenuation of renal tissue injury. Taken together, these results demonstrated that paeonol can prevent the renal toxic effects of cisplatin.

## 1. Introduction

Cisplatin (cis-diamminedichloroplatinum (II)) is a platinum compound that has revolutionized the treatment of various solid organ tumors. Because of its high effects, cisplatin is used for the treatment of a variety of malignancies, including testicular, head and neck, ovarian, cervical, non-small-cell lung carcinoma and many other types of cancer [1, 2]. However, nephrotoxicity, the most common adverse effect of cisplatin, often requires dose reduction or withdrawal of cisplatin [3]. Therefore, various studies have been investigated to reduce cisplatin-induced nephrotoxicity. 

Cisplatin-induced nephrotoxicity is related to direct tubular toxicity, inflammation, vascular factors, and oxidative stress [2]. Oxidative stress and inflammation have been suggested as the major mechanisms in the pathogenesis of cisplatin-induced nephrotoxicity [4].

Moutan Cortex, the root bark of *Paeonia suffruticosa Andrews*, is widely used in traditional medicine to treat various diseases including atherosclerosis, infection, and inflammation [5]. Previous studies have revealed that the extracts of Moutan Cortex can inhibit nitric oxide and tumor necrosis factor-alpha (TNF-*α*) in activated mouse peritoneal macrophages [5]. Further, our recent studies suggested that Moutan Cortex exerted recovery effects on cisplatin-induced nephrotoxicity *in vitro* [6]. In the present study, we searched whether extract of Moutan Cortex could ameliorate cisplatin-induced upregulation of serum creatinine and blood urea nitrogen (BUN) *in vivo*. After the confirmation that Moutan Cortex has the potential to attenuate the nephrotoxicity of cisplatin, we hypothesized that paeonol, a major phenolic component of Moutan Cortex, could attenuate cisplatin-induced renal damage. Recent studies have suggested that paeonol has various biological activities such as antiaggregatory, antioxidant, anxiolytic-like, and anti-inflammatory functions [7–9]. Moreover, it has been shown that natural antioxidants and anti-inflammatory agents such as curcumin, licorice extract, and *Zingiber officinale* can attenuate cisplatin-induced nephrotoxicity [10, 11]. In this study, we investigated whether paeonol attenuates cisplatin-induced renal damage by inhibiting inflammation and nitrosative stress.

## 2. Materials and Methods

### 2.1. Animals

Balb/c male mice (6 to 8 wk of age, weighing 20 to 25 g) were purchased from Orient Bio (Seongnam, Republic of Korea). All mice were kept under pathogen-free conditions with air conditioning and a 12 h light/dark cycle and had free access to food and water during the experiments. The study was approved by the Animal Care and Use Committee of Kyung Hee University.

### 2.2. Materials

Moutan Cortex extract was purchased from Sun Ten (Sun Ten Pharmaceutical Co., Ltd., Taiwan) and dissolved in distilled water to give a final concentration of 10% (w/v). The herb suspension supernatant (HSS) was then obtained by centrifugation at 300 ×g for 10 min at 4°C. Next, the HSS was passed through a sterile 0.20 *μ*m pore size filter unit (Sartorius AG, Germany). Paeonol was purchased from Wako (Wako Pure Chemical Industries, Ltd., Japan) and dissolved in phosphate buffered saline (PBS) at a concentration of 1 mg/ml. Cisplatin (*cis*-diammineplatinum II dichloride; Sigma-Aldrich, St. Louis, MO) was dissolved in 0.9% saline at a concentration of 1 mg/ml. Paeonol is one of many active main compounds contained in Moutan Cortex. Our previous study showed that 1 g of Moutan Cortex contained 0.46 ± 0.01 mg of paeonol [5].

### 2.3. Experimental Protocol

Each group of mice was administered Moutan Cortex (300 mg/kg body wt) or paeonol (20 mg/kg body wt) orally for three days. The control groups were only administered PBS. At day 4, the mice were given a single i.p. injection of either cisplatin (30, 20, or 10 mg/kg body wt) or an equal volume of saline. Mice were sacrificed at 48 or 72 h after the cisplatin administration for further evaluation.

### 2.4. Assessment of Renal Function and Histologic Examination

Blood samples were obtained from mice 0, 24, 48, and 72 h after cisplatin injection. Renal and liver function was assessed based on the BUN, creatinine, alanine aminotransferase, and aspartate aminotransferase, which were measured using a Fuji DRI-CHEM 3500i (Fuji Photo Film, Ltd., Japan). Kidney tissue was fixed in 4% paraformaldehyde and then embedded in paraffin, after which it was cut into 5 *μ*m sections and stained with hematoxylin and eosin. Three pathologists who were blinded to the experiments scored the degree of tubular injury. Renal tubular injury was assessed using a semiquantitative score in which the percentage of cortical tubules showing epithelial necrosis was assigned a score of either 0, none; 1, <10%; 2, 10–25%; 3, 25–75%; or 4, >75% [12].

### 2.5. Kidney Proinflammatory Cytokines

To examine the proinflammatory cytokines after cisplatin administration, the levels of TNF-*α* and IL-1*β* were measured in the kidneys by enzyme linked immunosorbent assay (ELISA; BD Biosciences, USA). Briefly, snap-frozen kidney tissue was homogenized in a PRO-PREP protein extraction solution (iNtRON Biotechnology, Inc., Korea), after which it was incubated for 20 minutes in ice and then centrifuged at 13,000 rpm (4°C) for 15 minutes. The supernatant was then used for kidney proinflammatory cytokine detection, which was accomplished using a BCA^TH^ Protein Assay Kit (Thermo Scientific, USA). The protein levels of cytokines were corrected for the total amount of protein, and the results were expressed as pg/mg.

### 2.6. Nitrite Estimation

Tissue nitrite was estimated using Greiss reagent and served as an indicator of NO production. Briefly, 500 *μ*L of Greiss reagent (1 : 1 solution of 1% sulfanilamide in 5% phosphoric acid and 0.1% naphthylamine diamine dihydrochloric acid in water) was added to 100 *μ*L of  kidney homogenates. The nitrite concentration was calculated using a standard curve for sodium nitrite. Nitrite levels were expressed as *μ*M/mg.

### 2.7. Statistical Analysis

Statistical analysis of the data was conducted using Prism 4.02 software (GraphicPad Software Inc., USA). All data are presented as the means ± S.E.M. The significance of differences between the experimental groups and the control was assessed by a Student's *t*-test. Kaplan-Meier analysis was used for the mouse survival analyses. For multiple comparison, two-way ANOVA was conducted. A *P* value <0.05 was taken to indicate significance.

## 3. Results

To ascertain whether paeonol could attenuate cisplatin-induced renal damage, we analyzed survival rate, serum creatinine, BUN, histologic change, proinflammatory cytokines, and nitrite production in cisplatin-injected mice.

### 3.1. Effects of Moutan Cortex on Cisplatin-Induced Renal Dysfunction

**Figure 1:**
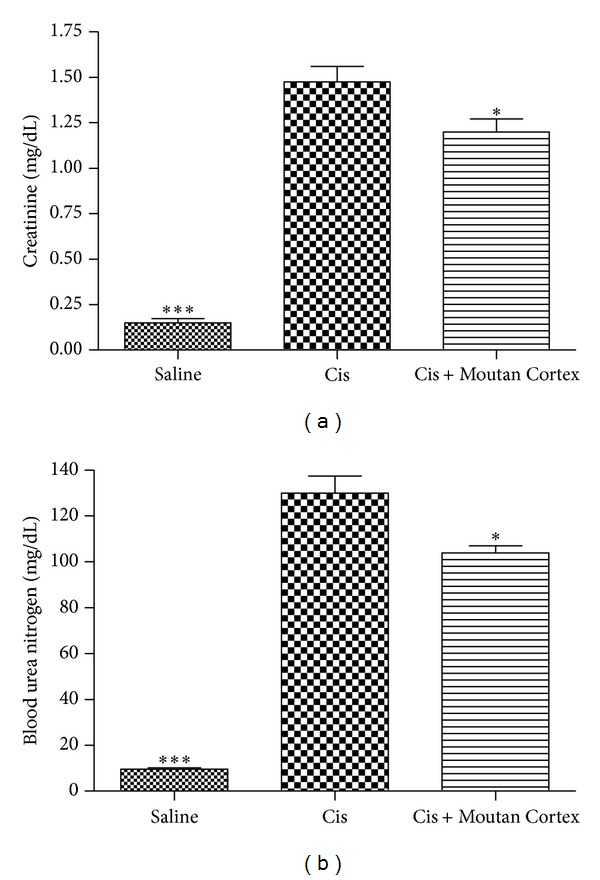
Effects of Moutan Cortex on creatinine and BUN production. All mice received a single dose of cisplatin intraperitoneally (30 mg/kg body wt). Serum creatinine (a) and BUN (b) were measured at 48 h after cisplatin injection. Saline: saline alone; Cis: cisplatin alone; Cis + Moutan Cortex: Moutan Cortex pretreatment and cisplatin treatment. Values shown are the mean ± S.E.M. Data were analyzed by a Student's *t*-test (*P < 0.05, ***P < 0.001 versus Cis; *n* = 4).

Both levels of the serum creatinine and BUN in cisplatin group were significantly increased at 48 h after cisplatin administration when compared with the saline group. The serum creatinine level in Moutan Cortex pretreatment group was significantly decreased at 48 h after cisplatin administration when compared with the cisplatin group (Figure 1(a)). In addition, BUN level was slightly reduced in Moutan Cortex pretreatment group at 48 h after cisplatin injection when compared with the cisplatin group (Figure 1(b)).

### 3.2. Effects of Paeonol on Kidney and Liver Dysfunction

**Figure 2:**
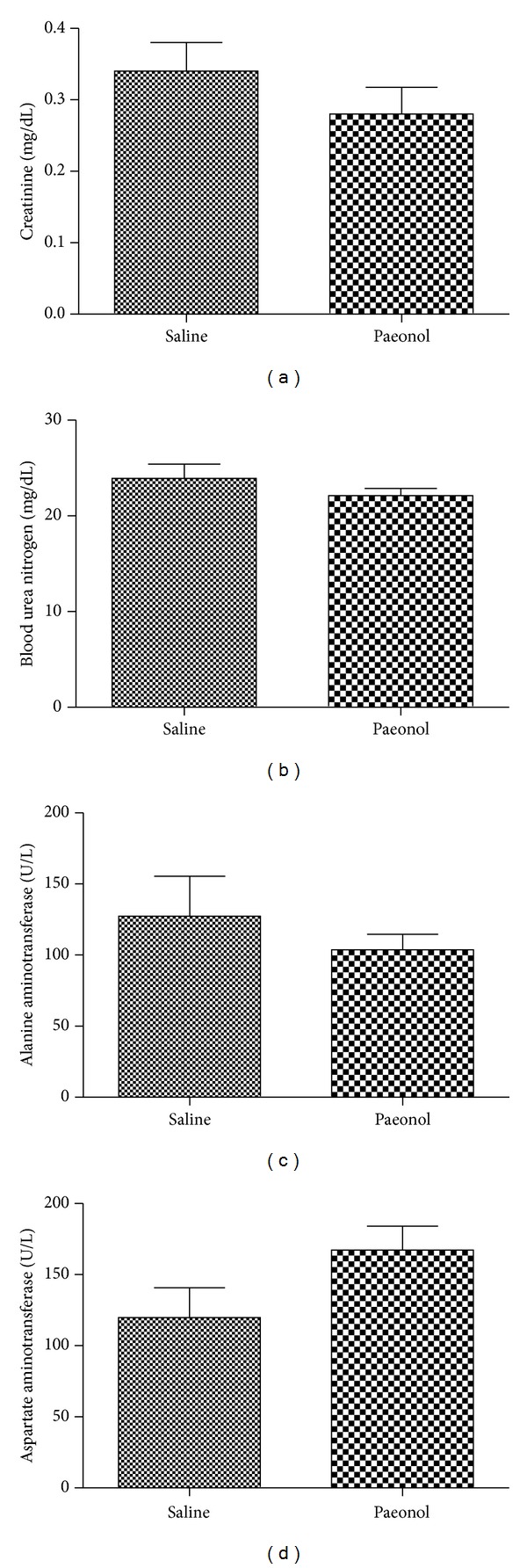
Effects of paeonol on kidney and liver dysfunction. Each group of mice was administered paeonol (20 mg/kg body wt) or saline via a single intraperitoneal injection for three days. At day 4, blood samples were obtained from mice to check the toxicity of paeonol. Serum creatinine (a), BUN (b), alanine aminotransferase (c), and aspartate aminotransferase (d) were measured. Values shown are the mean ± S.E.M. Data were analyzed by a Student's *t*-test (*n* = 5).

Each group of mice was administered paeonol (20 mg/kg body wt) or saline via a single intraperitoneal injection for three days. At day 4, blood samples were obtained from mice to check the toxicity of paeonol. The dose of 20 mg/kg paeonol caused no harmful effects on kidney and liver dysfunction (Figure 2).

### 3.3. Effects of Paeonol on Survival after Cisplatin Administration

**Figure 3:**
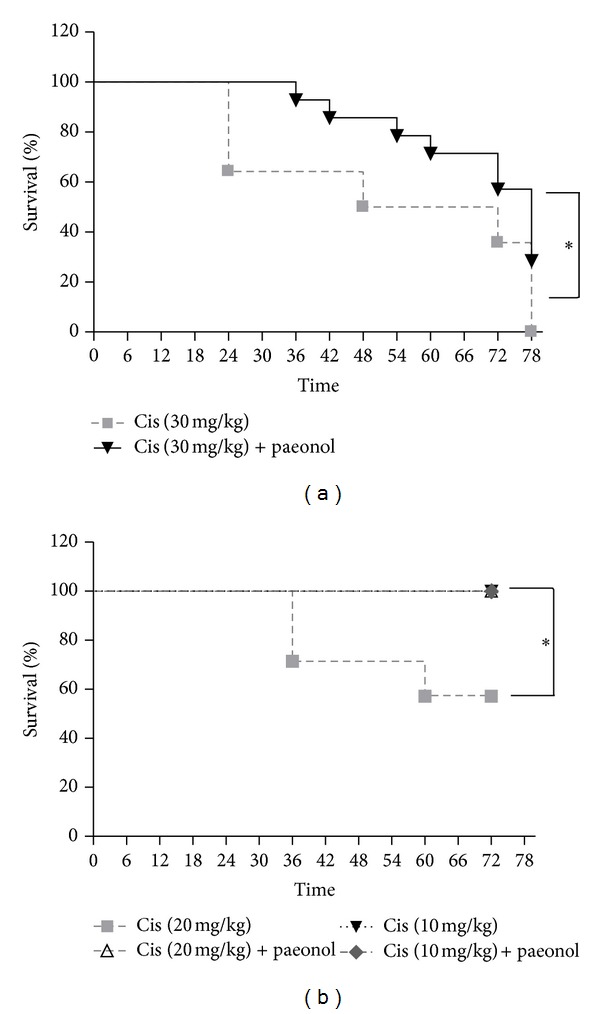
Survival in cisplatin-treated mice and paeonol-pretreated mice. (a) All mice received a single dose of cisplatin intraperitoneally (30 mg/kg body wt) and were followed up to 78 h. Compared with a 60 median survival time in cisplatin-treated group, paeonol-pretreated group had a 78 median survival time. Data were analyzed by a Kaplan-Meier test (*P < 0.05 versus Cis; *n* = 14). (b) Mice received cisplatin intraperitoneally (20 or 10 mg/kg body wt) and were followed up to 72 h. Data were analyzed by a Kaplan-Meier test (*P < 0.05 versus Cis; *n* = 7).

Both the paeonol-pretreated group and cisplatin group received three different doses of cisplatin (30, 20, or 10 mg/kg body wt) and were followed up for 78 h. At 78 h after injection of 30 mg/kg cisplatin, all of the cisplatin group mice had expired. However, the paeonol-pretreated mice had 28% survival. Moreover, at 72 h after 20 mg/kg of cisplatin administration, all of the paeonol-pretreated mice survived, but 42% of cisplatin group mice had expired. Taken together, these results show that pretreatment with paeonol improved survival after cisplatin administration in mice (Figure 3).

### 3.4. Effects of Paeonol on Cisplatin-Induced Renal Dysfunction

**Figure 4:**
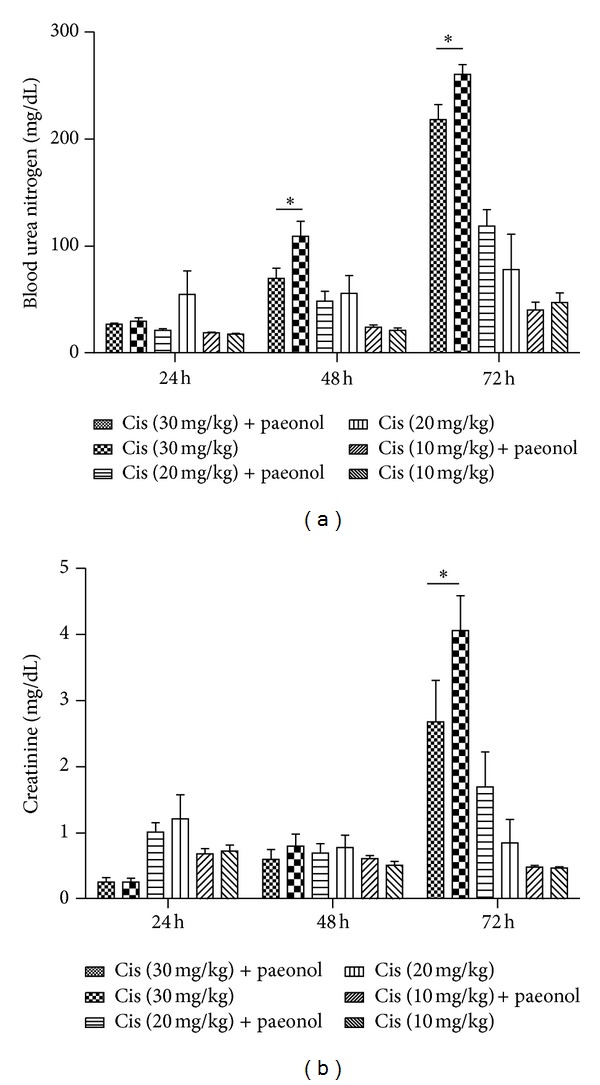
Effects of paeonol on cisplatin-induced renal dysfunction. Serum creatinine (a) and BUN (b) were measured at 24, 48, and 72 h after cisplatin injection (30, 20, or 10 mg/kg body wt). Data were analyzed by a two-way ANOVA test (*P < 0.05 versus Cis (30 mg/kg); *n* = 7).

The serum creatinine levels were significantly higher in the cisplatin group at 72 h after administration of 30 mg/kg of cisplatin when compared with the paeonol pretreatment group (Figure 4(a)). BUN level was also remarkably reduced in the paeonol pretreatment group at 48 h and 72 h after 30 mg/kg of cisplatin injection when compared with the group that only received cisplatin (Figure 4(b)). In 20 mg/kg of cisplatin-administered mice, creatinine and BUN levels were slightly decreased at 72 h by paeonol pretreatment. Besides, creatinine and BUN levels were not increased by 10 mg/kg of cisplatin treatment as previously reported [13, 14].

### 3.5. Effects of Paeonol on Cisplatin-Induced Renal Injury

**Figure 5:**
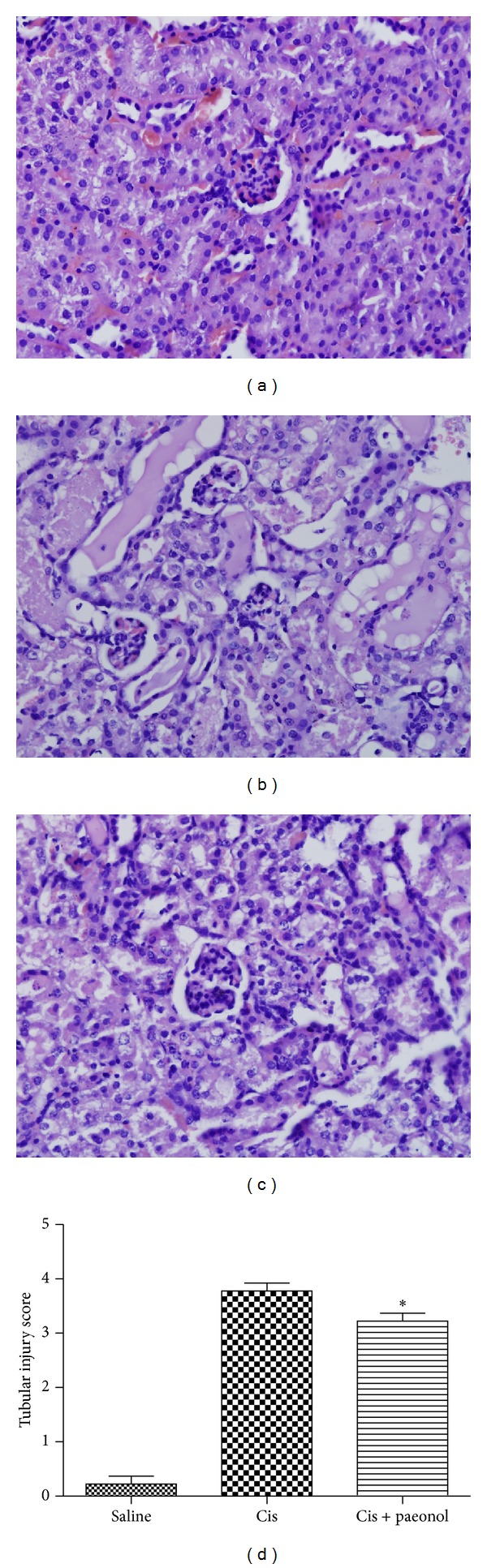
Effects of paeonol on renal histology in mice. Sections of  kidney were stained with H&E 72 h after cisplatin injection. Saline treatment alone (a); cisplatin treatment alone (b); paeonol and cisplatin treatment (c). Renal tubular injury was assessed using a semiquantitative score in which the percentage of cortical tubules showing epithelial necrosis was assigned a score of either 0, none; 1, <10%; 2, 10–25%; 3, 25–75%; or 4, >75% (d). Saline: saline alone; Cis: cisplatin alone; Cis + paeonol: paeonol pretreatment and cisplatin treatment (*P < 0.05 versus Cis, *n* = 3).

The degree of renal tubular injury in the control- and paeonol-treated groups was observed at 72 h after cisplatin administration. Paeonol-treated groups showed slightly less renal injury than mice that received saline (Figure 5).

### 3.6. Effects of Paeonol on Cisplatin-Induced Proinflammatory Cytokines

**Figure 6:**
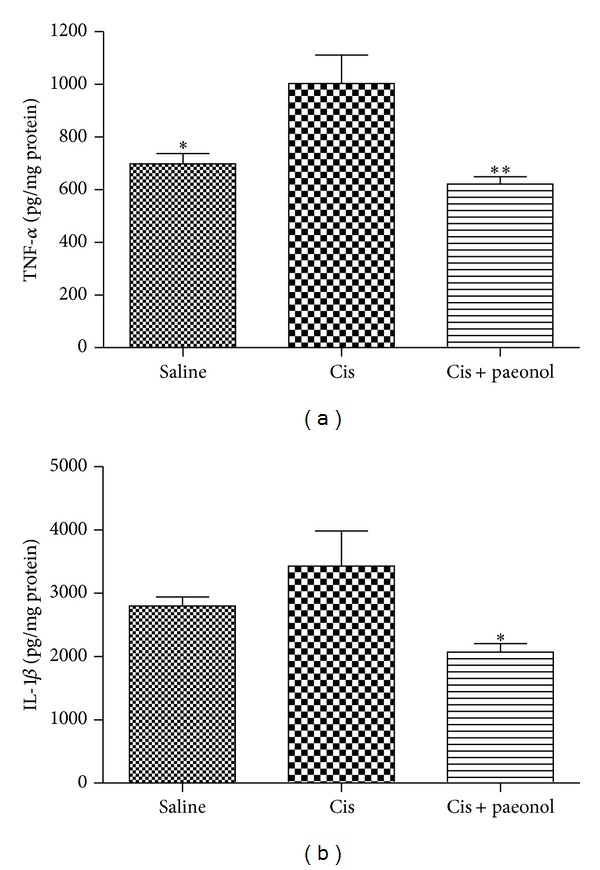
Effects of paeonol on cisplatin-induced proinflammatory cytokines. Kidneys were obtained at 72 h after cisplatin injection, and cytokine levels of TNF-*α* (a) and IL-1*β* (b) were determined by ELISA. Saline: saline alone; Cis: cisplatin alone; Cis + paeonol: paeonol pretreatment and cisplatin treatment. Values shown are the mean ± S.E.M. Data were analyzed by a Student's *t*-test (*P < 0.05, **P < 0.01 versus Cis; *n* = 4 for saline; *n* = 5 for Cis or Cis + paeonol).

To investigate the proinflammatory molecules generated by cisplatin renal injury, the cytokine levels of  TNF-*α* and IL-1*β* were measured at 72 h after cisplatin administration. Cisplatin-treated mice had significantly increased levels of  TNF-*α* at 72 h after cisplatin injection, while paeonol pretreatment reduced expression of  TNF-*α* in the kidney. IL-1*β* was increased by cisplatin treatment, but its increasing was not significant. However, paeonol significantly reduced IL-1*β* production by cisplatin treatment (Figure 6).

### 3.7. Effects of Paeonol on Cisplatin-Induced Nitric Oxide Production

**Figure 7:**
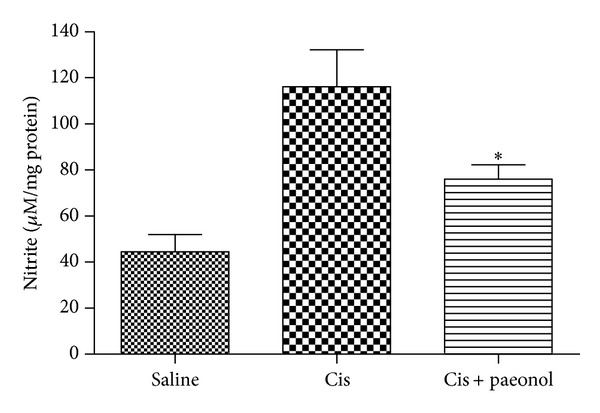
Effects of paeonol on cisplatin-induced nitric oxide production. Nitrate levels in kidney tissue were measured in control, cisplatin, and paeonol groups after a single dose of cisplatin (30 mg/kg body wt). Saline: saline alone; Cis: cisplatin alone; Cis + paeonol: paeonol pretreatment and cisplatin treatment. Values shown are the mean ± S.E.M. Data were analyzed by a Student's *t*-test (*P < 0.05 versus cis; *n* = 4 for saline; *n* = 5 for Cis or Cis + paeonol).

Nitrite levels of kidney tissue homogenates were measured at 72 h after cisplatin injection. Nitrite levels of kidney tissue were significantly elevated by cisplatin administration. The nitrite production in kidney tissue was slightly attenuated in paeonol-pretreated group compared with control group (Figure 7).

## 4. Discussion

Nephrotoxicity is a major side effect that limits the use of cisplatin in many cancer patients. Indeed, many studies have documented that apoptosis/necrosis, reactive oxygen species, and inflammation play a major role in the pathogenesis of cisplatin-induced acute renal failure [15, 16]. Therefore, various studies have been conducted to reduce the cisplatin-induced nephrotoxicity. Recent studies have suggested that natural antioxidants and anti-inflammatory agents such as curcumin, licorice extract, and *Zingiber officinale* can attenuate cisplatin-induced nephrotoxicity [10, 11]. Additionally, proinflammatory cytokines such as TNF-*α*, IL-6, and IL-1*β* have been shown to be associated with cisplatin-induced acute renal failure [17–19].

Moutan Cortex, the root bark of *Paeonia suffruticosa Andrews*, has been used extensively as a traditional medicine for treatment of various diseases such as atherosclerosis, infection, and inflammation. Previous studies have revealed that the extracts of Moutan Cortex can inhibit nitric oxide and TNF-*α* in activated mouse peritoneal macrophages [5]. 

A variety of compounds including paeonoside, paeonolide, apiopaeonoside, paeoniflorin, oxypaeoniflorin, benzoyloxypaeoniflorin, benzoylpaeoniflorin, paeonol, and sugars have been identified in Moutan Cortex [20]. Paeonol, a major phenolic component of Moutan Cortex, has various biological activities such as antiaggregatory, antioxidant, anxiolytic-like, and anti-inflammatory functions [8]. In this study, paeonol treatment significantly reduced the elevated levels of serum creatinine and BUN. 

In addition, the role of proinflammatory cytokines in cisplatin-induced acute renal failure has been well documented [17, 18], and elevation of the proinflammatory cytokines TNF-*α* and IL-1*β* as well as that of IL-6 has been demonstrated in humans with acute renal failure [21]. Given that recent research has demonstrated that TNF-*α* plays a significant role in cisplatin-induced nephrotoxicity, we measured several proinflammatory cytokine levels in kidney tissue. We found that cisplatin administration increased the kidney tissue TNF-*α* level, whereas paeonol pretreatment led to a significant decrease in the TNF-*α* level. The increase in IL-1*β* level was also attenuated in paeonol-pretreated mice. Taken together, these results indicate that paeonol may reduce the production of TNF-*α* and IL-1*β* cytokines that is due to the direct anti-inflammatory effects of paeonol.

Nitric oxide (NO) is recognized as a mediator and regulator of inflammatory responses. NO is involved in host defense with cytotoxic properties against pathogenic microbes, but high levels of NO production in response to inflammatory stimuli can cause proinflammatory and destructive effects on host tissue [22]. Cisplatin treatment also causes significant increases in the activity of calcium-independent nitric oxide synthase (NOS) in rat kidney and liver, which leads to an increase in serum NO levels as well as tissue NO formation [23, 24]. Jung et al. reported that the upregulation of oxide mNOS and peroxynitrite formation in cisplatin treatment are key events that influence the development of harmful parameters that occur during cisplatin-associated kidney failure [25]. It is well known that selective iNOS inhibition reduces cisplatin-induced nephrotoxicity and nitrosative stress [26]. In the present study, paeonol treatment prevented the increases in kidney tissue nitric oxide when compared with cisplatin-treated mice. Recently, paeonol treatment was found to inhibit iNOS protein expression induced by TNF-*α* and iNOS mRNA expression *in vitro* [8, 27]. Therefore, our results indicate that paeonol has the potential to reduce nitrosative stress in cisplatin-induced nephrotoxicity. 

In conclusion, our study provides evidence that Moutan Cortex has a potential to attenuate nephrotoxicity of cisplatin and its major constituent paeonol can be a therapeutic intervention for cisplatin-induced renal injury. However, what molecular mechanisms are related to the protective effects of paeonol on kidney and the influences of Moutan Cortex and paeonol on the therapeutic efficacy of cisplatin should be further studied.
